# A Novel Wearable Forehead EOG Measurement System for Human Computer Interfaces

**DOI:** 10.3390/s17071485

**Published:** 2017-06-23

**Authors:** Jeong Heo, Heenam Yoon, Kwang Suk Park

**Affiliations:** 1 Interdisciplinary Program of Bioengineering, Seoul National University, Seoul 03080, Korea; hjeong20@bmsil.snu.ac.kr (J.H.); hnyoon@bmsil.snu.ac.kr (H.Y.); 2 Department of Biomedical Engineering, College of Medicine, Seoul National University, Seoul 03080, Korea

**Keywords:** human computer interface, HCI, electrooculogram, EOG, forehead, eye movement

## Abstract

Amyotrophic lateral sclerosis (ALS) patients whose voluntary muscles are paralyzed commonly communicate with the outside world using eye movement. There have been many efforts to support this method of communication by tracking or detecting eye movement. An electrooculogram (EOG), an electro-physiological signal, is generated by eye movements and can be measured with electrodes placed around the eye. In this study, we proposed a new practical electrode position on the forehead to measure EOG signals, and we developed a wearable forehead EOG measurement system for use in Human Computer/Machine interfaces (HCIs/HMIs). Four electrodes, including the ground electrode, were placed on the forehead. The two channels were arranged vertically and horizontally, sharing a positive electrode. Additionally, a real-time eye movement classification algorithm was developed based on the characteristics of the forehead EOG. Three applications were employed to evaluate the proposed system: a virtual keyboard using a modified Bremen BCI speller and an automatic sequential row-column scanner, and a drivable power wheelchair. The mean typing speeds of the modified Bremen brain–computer interface (BCI) speller and automatic row-column scanner were 10.81 and 7.74 letters per minute, and the mean classification accuracies were 91.25% and 95.12%, respectively. In the power wheelchair demonstration, the user drove the wheelchair through an 8-shape course without collision with obstacles.

## 1. Introduction

Human computer/machine interface (HCI/HMI) is an interfacing technology between the user and a computer/machine. In particular, HCIs using bio-signals that are voluntarily controlled, such as electromyograms (EMGs), electroencephalograms (EEGs), and electro-oculograms (EOGs), have been studied for people with disabilities. Artificial prosthesis and communication systems, including virtual keyboards, are typical HCI applications [1,2,3]. HCIs are very important technologies for people with disabilities because they are not just support tools, but they could improve a disabled person’s quality of life.

Amyotrophic lateral sclerosis (ALS), well known as Lou Gehrig’s disease, is a disease that causes the death of motor neurons [4]. In early ALS, the muscles of the hands, fingers, and legs become weak and thin. Additionally, it becomes difficult to talk and swallow food. As time passes, the symptoms get worse until voluntary breathing becomes difficult in the end stage. In many cases, eye movements are usually spared until the final stage, although the limbs and tongue are paralyzed. Thus, many patients with ALS use communication supporting tools based on eye movement [5].

Interface systems using brain activities, such as EEGs, Electro-corticograms, and magneto-encephalograms, are called brain–computer interfaces (BCIs). Steady-state visual evoked potential (SSVEP) or P300-based BCI systems for communication support have been reported by many BCI research groups. Valbuena et al. introduced the Rhombus layout SSVEP-based virtual keyboard using five flickering stimuli [6]. They arranged the letters according to on their prevalence in the English language. The P300 Speller described by Farwell and Donchin provided a 6 × 6 matrix of characters [7]. They used the P300 component of the event-related potential (ERP) elicited by rarely occurred “attended” stimuli.

An EOG is a signal that measures the potential difference between the retina and the cornea [8]. The eyeball can be modeled as a dipole with the positive cornea in the front and the negative retina in the back. The primary applications of EOG are ophthalmological diagnosis and eye movement recording. To record eye movements, surface electrodes are typically placed at four positions around the eyes (above, below, left, and right). The pair of electrodes placed above and below the eyes is used to measure the vertical movement of the eyes, and the pair of electrodes placed to the left and right of the eyes are used to measure the horizontal movement of the eyes. When the gaze is shifted to the left side, the electrical field of the left side of the eyes becomes positive and the opposite side becomes negative.

A virtual keyboard based on EOG was introduced by Tecce et al. in 1998 [2]. The results of studies on this keyboard showed its usefulness as a communication assisting system for those who are paralyzed or unable to speak. In 2002, Barea et al. developed a wheelchair driving system using EOG to increase the mobility of disabled users who have motor paralysis [9]. Usakli et al. reported an EOG measurement system using an opposite phase summing approach to remove the direct current (DC) level and reduce power line noise in 2010 [10]. Using the nearest neighbor algorithm, they classified eye movement in four directions and two types of blinking with 95% accuracy. In addition, there are several studies that developed goggle-type wearable EOG measurement systems to improve the user’s comfort by reducing preparation time [11,12,13]. However, the electrodes around the eyes often cause discomfort to the user, and this discomfort has been the motivation for many studies [14,15,16,17]. To simplify and improve user convenience, many studies have proposed new electrode placement that can replace conventional EOG electrode placement [14,15,16,17,18].

The aim of this study was to measure eye movements by placing a small number of electrodes only on the forehead for wearable HCI system. Three electrodes were placed on the forehead in the vertical and horizontal directions, sharing a positive electrode, and the ground electrode was placed at the center of the forehead. We developed a headband-type wireless wearable sensor system for detection of six class of eye movements and verify it through three applications.

## 2. Materials and Methods

### 2.1. Placement of Electrodes and Comparison to Conventional EOG

**Figure 1:**
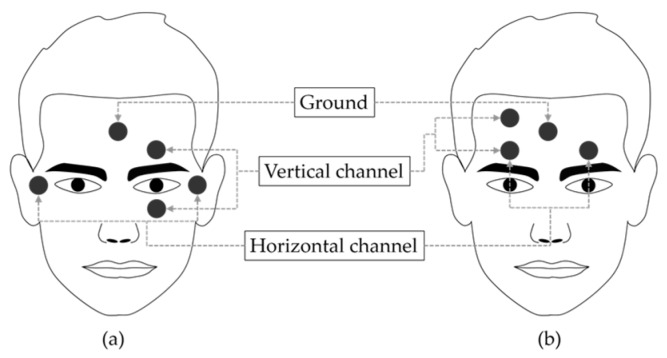
Electrode placement for (**a**) conventional EOG and (**b**) proposed forehead EOG.

In most of the previous EOG-based HCI studies, electrodes were placed such that one was located both above and below the eye (left or right) to measure vertical eye movement and one was located both at the left and right of the outer canthi to measure horizontal eye movement (Figure 1a). The novel electrode placement proposed in this study is shown in Figure 1b.

The positive electrode for the vertical and horizontal channels was shared, and it was placed above the eye. The negative electrode for the vertical channel was placed above the positive electrode. The negative electrode for the horizontal channel was placed above the other eye. We evaluated the usability of the proposed electrode positioning with a commercial EOG measurement system (EOG100C, Biopac Systems Inc., Goleta, CA, USA). The gain was set to 5000 and the frequency bandwidth was set to 0.05–35 Hz.

### 2.2. Wearable System Design

**Figure 2:**
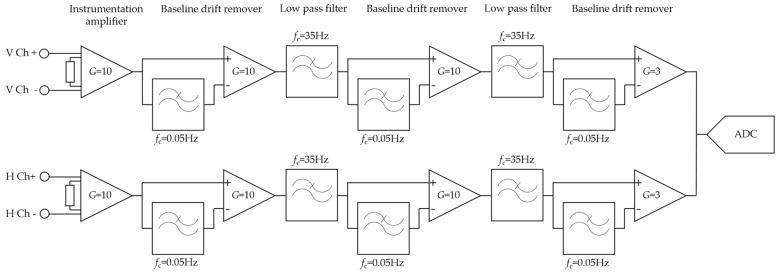
Analog circuit block diagram for forehead EOG measurement system.

**Figure 3:**
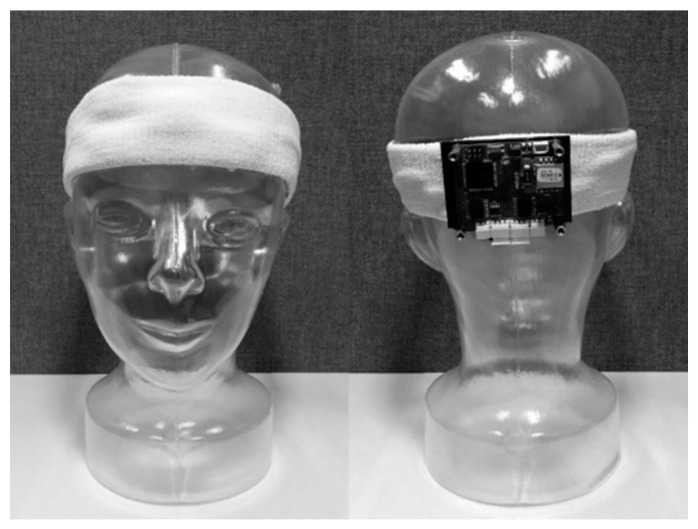
Headband-type wearable forehead EOG measurement system.

We designed a wearable forehead EOG system based on headband. The Ag/AgCl electrodes were placed inside the head band according to the arrangement of Figure 1b. Analog filtering and amplifying circuit was developed with reference to the study of Usakli et al. [10]. To measure vertical and horizontal eye movements, two circuit channels were required. Each channel was composed of an instrumentation amplifier, three DC drift removers with gain, and two low-pass filters. The DC drift remover was composed of a low-pass filter and differential amplifier. All filters were 2-order, Sallen-Key Butterworth filters. The total gain was set to 3000 and the bandwidth was set to 0.05–35 Hz. The schematic of the analog stages is shown in Figure 2. After analog filtering and amplifying, the signal was converted to digital data with a 16-bit resolution and a 256 sampling frequency. Converted data were transmitted wirelessly by Bluetooth 2.0. The system was powered by a rechargeable 3.7V Li-polymer battery (H703448, Power Source Energy Co., Taipei, Taiwan). The operation time of the system was approximately 18 hours with a fully charged 1250 mA∙h battery. The system was not designed with an objective of optimizing operating time; this could be increased by using low-power chips and processor. The entire system was integrated on a 44 mm × 55 mm printed circuit board and placed on the back side of a headband. Figure 3 shows images of the headband-type forehead EOG measurement system.

### 2.3. Signal Processing and Eye Movement Classification

**Figure 4:**
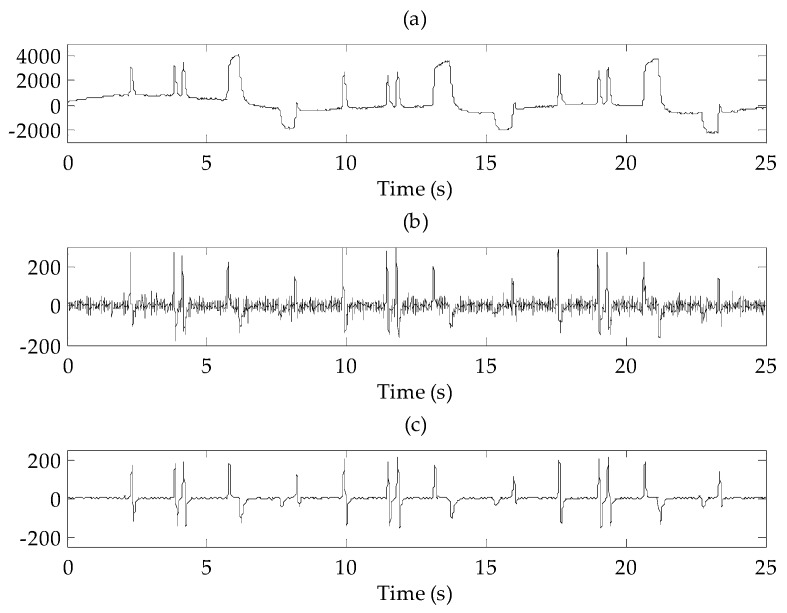
(**a**) Recorded signal of a vertical forehead EOG channel; (**b**) Derivative signal of (**a**); (**c**) 10 Hz low-pass filtered signal of (**b**).

Baseline drift is a critical limitation of forehead EOG, as shown Figure 4 in result section. The signal was differentiated to eliminate the baseline, which drifted with a low frequency. After differentiation, the signal was low-pass filtered with a cutoff frequency of 10 Hz to remove high-frequency noise. Figure 4 shows the processed signal of the vertical channel. As shown in Figure 4c, finally, baseline drift was eliminated and the specific eye movement patterns were observed.

**Figure 5:**
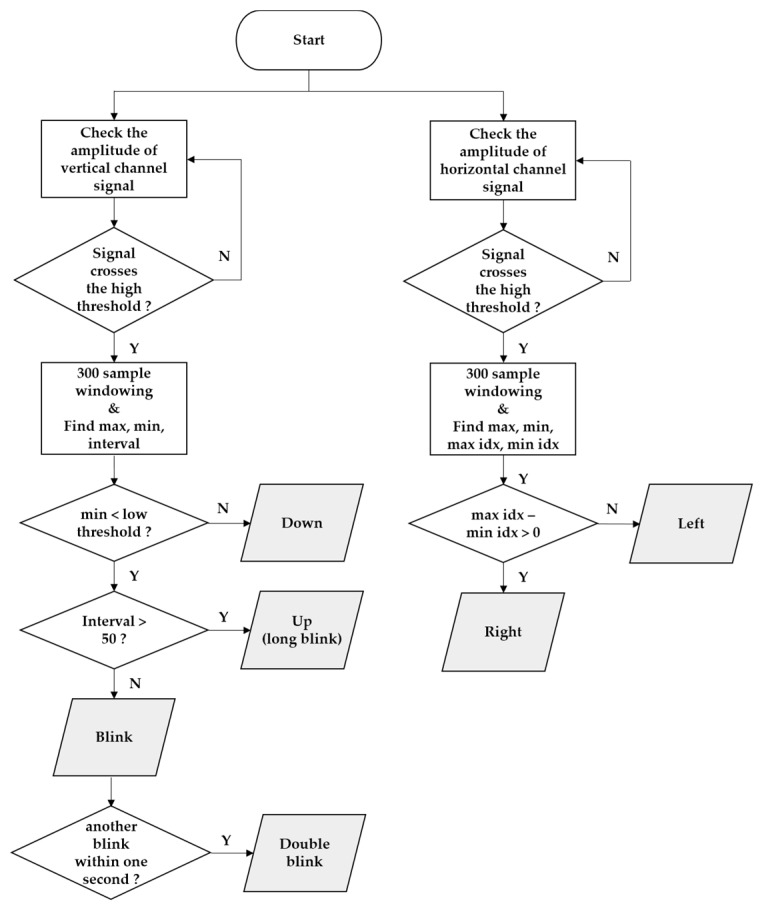
Flowchart of the eye movement classification algorithm.

The real-time classification algorithm was developed based on the patterns that were generated by eye movement. Figure 5 shows a flow chart of the eye movement classification algorithm. When a positive peak that was higher than the threshold occurred, 300 samples were extracted, centered on the peak. In the extracted window, the maximum and minimum values and their time indexes were extracted as features that were used for classifying the eye movement directions. Six types of eye movements were classified by the algorithm (up, down, left, right, blink, and double blink). Blinks that occurred twice within 1 s were classified as a ‘double blink’. Single blinks were detected in the algorithm but were not encoded to any action in the application to avoid errors caused by involuntary blinking. The upper and lower thresholds of each channel were optimized according to individual EOG characteristics.

### 2.4. Application

#### 2.4.1. Virtual Keyboard

To evaluate usability of the newly developed forehead EOG measurement system, we employed two types of virtual keyboards. First, a modified Bremen BCI speller was employed, which is widely used for steady state visual evoked potential (SSVEP)-based brain computer interface studies. This virtual keyboard was programmed using Microsoft Visual Studio 8.0. Five classes of eye movements were used for typing instead of SSVEP stimulations. At the start of typing, the cursor was placed over the letter “E”, which is at the center of the virtual keyboard layout. Users could move the cursor step by step using their own eye movements. Then, users could select a letter by “double blinking”. To avoid false signaling caused by involuntary blinking, double blinking was used to select the desired letter. When the letter was selected, the cursor returned to ‘E’ automatically, and the selected letter was typed below the keyboard layout.

The second type virtual keyboard was based on automatic sequential row-column scanning for severe ALS patients who cannot control their eye movements. The scanning method is also widely used as a communication tool for LIS patients. Automatic scanning is used in cases where the user has only one way to signal the interface. In this case, a double blink was used as a switch signal. We developed a 5 × 6 matrix virtual keyboard including 26 letters (“a” to “z”), space, period, delete, and clear. Typing started by first scanning each row. The red cursor automatically shifted to the next row after 2 s. When the cursor arrived at the row that included the target letter, the user selected the row with a double blink. Then, column scanning began using a blue cursor. Similar to row scanning, when the cursor arrived at the column that included the target letter, the user selected the column with another double blink. The target letter, which was located at the cross point of the red and blue cursors, was then typed above the matrix. The letters were arranged so that the most frequently used letters were located closest to the upper-left-hand corner.

Four healthy and normal vision subjects participated in the virtual keyboard experiments (four males; mean age: 26.75 ± 3.77 years). One subject (S1) was familiar with the system and the other subjects (S2~S4) had no experience. The inexperienced subjects had time to practice for about an hour and the experiments proceeded the next day.

#### 2.4.2. Power Wheelchair

**Figure 6:**
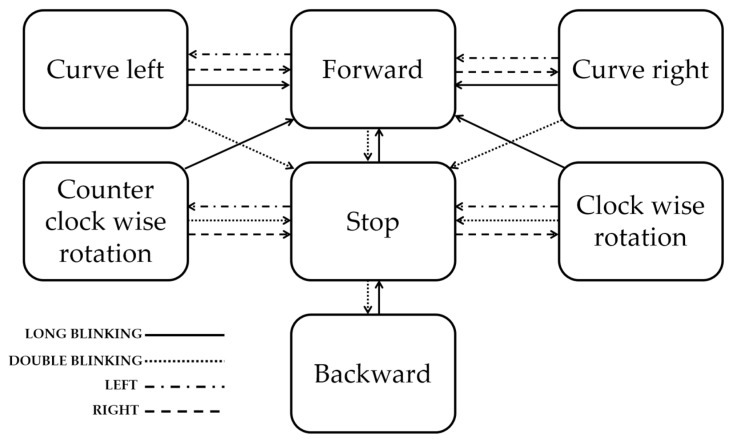
State diagram of the power wheelchair.

**Figure 7:**
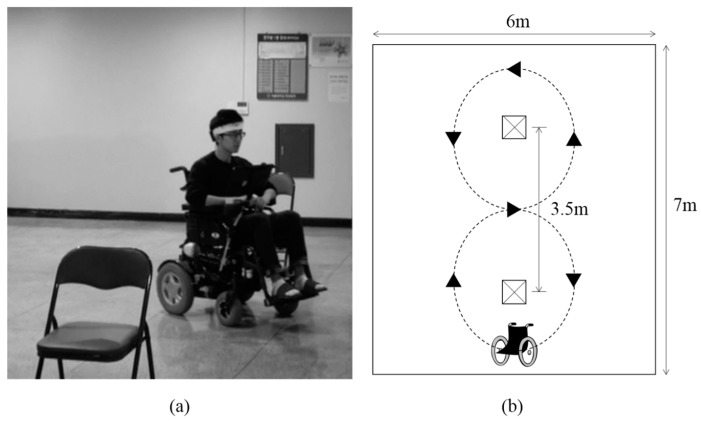
Wheelchair driving using wearable forehead EOG sensor. (**a**) A snapshot of wheelchair driving demonstration; (**b**) The wheelchair driving task route.

In addition to the virtual keyboards, we evaluated the usability of forehead EOG for driving a power wheelchair. In view of safety considerations, experienced subjects participated in the experiment. The commercialized power wheelchair was modified to drive using forehead EOG signals. The EOG signals measured by the headband sensor were filtered, amplified, and transmitted to a tablet PC wirelessly via Bluetooth. Then, a classification program classified the eye movements and transmitted the results to a custom-made digital–analog converting module that replaced the wheelchair’s joystick controller. Four types of eye movements were used as commands to change the state of the wheelchair. The state diagram of the power wheelchair is shown in Figure 6. Two obstacles were placed at the center of a 7 m × 6 m hall with 3.5 m spacing. The user’s task was to drive the wheelchair and avoid obstacles along the illustrated route as shown in Figure 7.

## 3. Results

### 3.1. Forehead EOG

**Figure 8:**
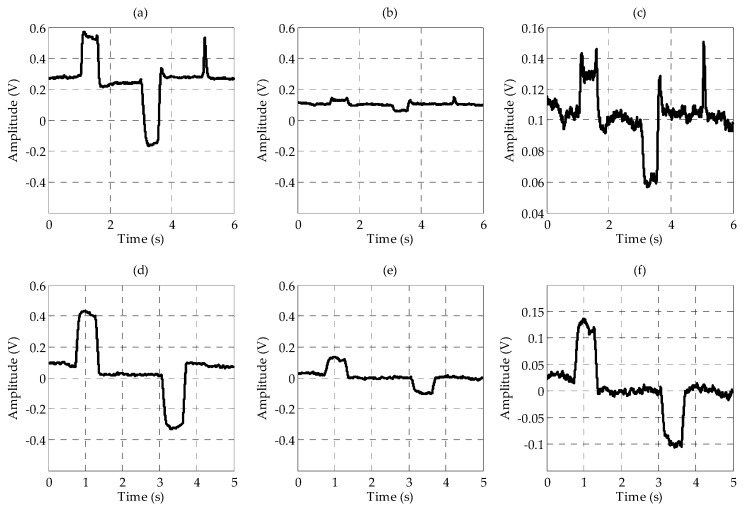
Comparison of conventional EOG and forehead EOG. (**a**) Vertical channel signal from conventional EOG; (**b**) vertical channel signal from forehead EOG; (**c**) magnified view of vertical channel signal from forehead EOG; (**d**) horizontal channel signal from conventional EOG; (**e**) horizontal channel signal from forehead EOG (**f**) magnified view of horizontal channel signal from forehead EOG.

Figure 8 shows an EOG signal obtained from both conventional electrode placement and the proposed forehead electrode positioning. Figure 8a,d shows the vertical and horizontal channel signals, respectively, from the conventional EOG. Figure 8c,f shows the magnified view of vertical and horizontal channel signals, respectively, from the forehead EOG. Although the amplitude and signal quality of the forehead EOG were lower than for the conventional EOG, amplitude variations due to eye movement were clearly observed.

**Figure 9:**
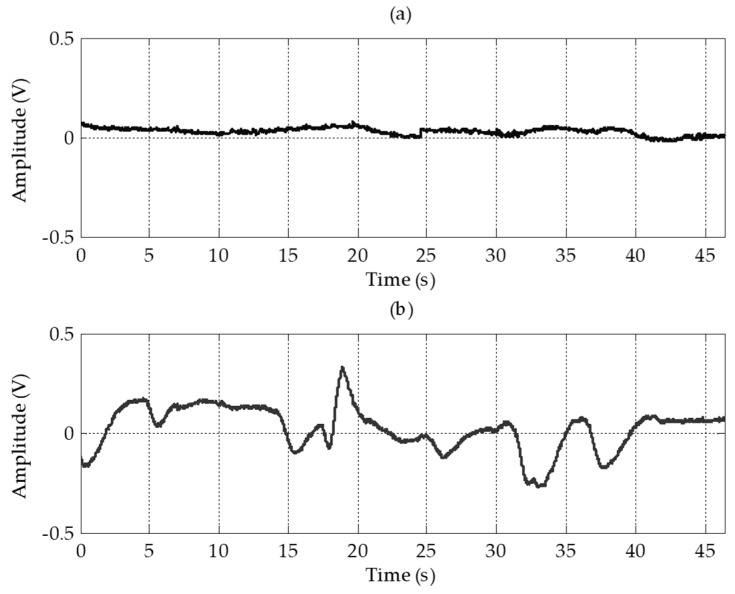
Baseline drift in the horizontal channel when user stared at center point. (**a**) Conventional EOG; (**b**) Forehead EOG.

Figure 9 shows a comparison of baseline drift between conventional and forehead EOG for the horizontal channel when a subject began gazing at a single point in front of them. Unlike other physiological signals, EOG has a baseline drift problem because of the low cutoff frequency of the high-pass filter. Moreover, in forehead EOG, more severe baseline drift occurred than in conventional EOG. To overcome this baseline drift problem, we applied the simple signal processing technique described in Section 2.3 before classifying eye movements.

**Figure 10:**
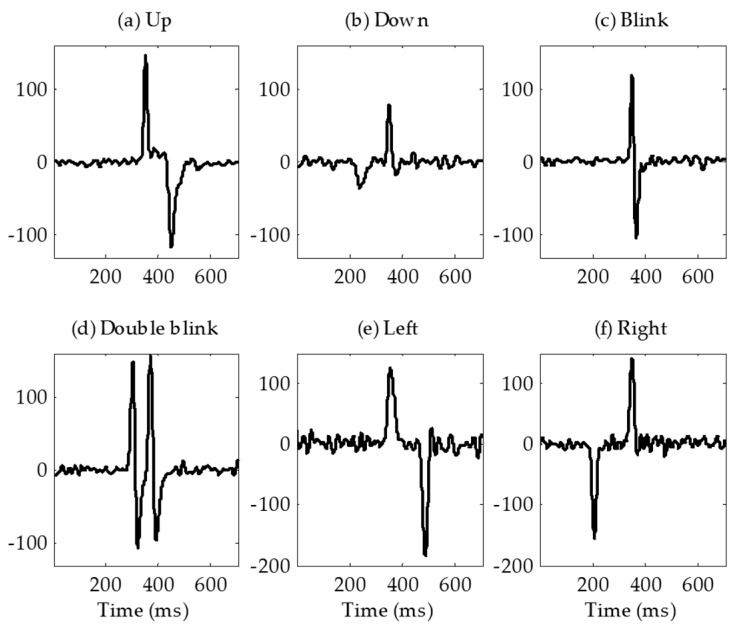
Forehead EOG signal patterns after signal processing. (**a**) Up (long blink); (**b**) Down; (**c**) Blink; (**d**) Double blink; (**e**) Left and (**f**) Right.

Figure 10 shows forehead EOG signal patterns after the signal processing. Figure 10a–f represents up (long blink), down, blink, double blink, left and right in that order. Figure 10a–d was captured in vertical channel signal, Figure 10e,f was captured in horizontal channel signal. The signal pattern of “down” had relatively low amplitude, especially negative peak. The reason may be that the distance between the sensor and the source is farther than the other movements, or the subject was unfamiliar with eye movements downward.

### 3.2. Application: Virtual Keyboards

Table 1 summarizes the accuracy and typing speed of the virtual keyboard demonstrations. The task words were “SEOUL NATIONAL UNIVERSITY” without word spacing. The minimum number of commands required to complete the task was 62. In the demonstration, the “up” eye movement was replaced by a “long blink” because the subjects complained that it was difficult to move their gaze upwards. Because a “long blink” generated almost the same signal pattern as “up”, the classification algorithm did not need to be modified. An error indicates that the cursor moved differently from the user’s intention. A failure indicates the user moved his eyes but the cursor did not move. For the modified Bremen keyboard demonstration, the mean accuracy was 91.25% and the mean typing speed was 10.81 letters per minute. For the automatic RC scanning keyboard demonstration, the mean accuracy was 95.12% and the mean typing speed was 7.75 letters per minute. Because the typing speed varied based on the composition of characters in the words and the user’s condition, the typing speed and accuracy were not proportional.

**Figure 11:**
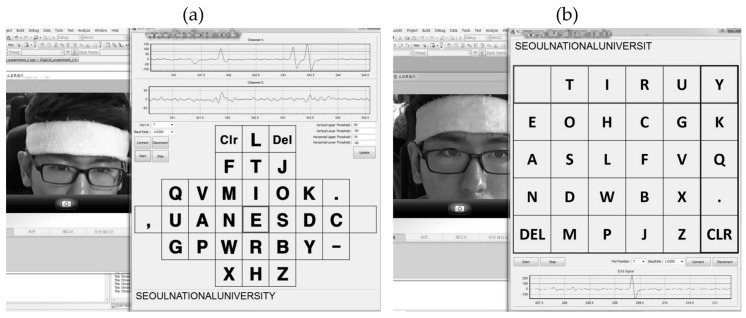
A snapshot of the virtual keyboard demonstration based on (**a**) the modified Bremen BCI speller; (**b**) the automatic sequential row-column scanning.

Figure 11a shows an image of the modified Bremen keyboard demonstration completed by S1. It took approximately 2 min and 39 s for a user to type these 23 letters. The typing speed was approximately 8.68 letters per minute. There were five failures to move the cursor and one error. The total number of trials was 70, which included a minimum of 62 commands to complete the task, three additional commands that were caused by the one error, and five failures. Thus, the classification accuracy was 91.43%. A demonstration video can be found at supplementary materials (Video S1) or https://youtu.be/Np_o1mxxaoY.

Figure 11b shows an image of a demonstration of the virtual keyboard based on automatic sequential row-column scanning by S1. A “double blink” was used as the command switch, which selected the row and column in turn. The task words were the same as in the previous demonstration. It took approximately 2 min and 59 s for a user to type these 23 letters. The speed of typing was approximately 7.71 letters per minute. There were no fails or errors. Thus, the classification accuracy was 100%. A demonstration video also can be found at supplementary materials (Video S2) or https://youtu.be/MmCkv-Oiiag.

### 3.3. Application: Power Wheelchair

The power wheelchair tasks included completing a figure “0” and a figure “8” route around the two obstacles. During the demonstration, the wheelchair’s status changed 19 times. The subject controlled the wheelchair through the arranged course while avoiding collision with the obstacles. A demonstration video of a user driving the power wheelchair can be found at supplementary materials (Video S3) or https://youtu.be/eveoN8Y8q-M.

## 4. Discussion

### 4.1. Forehead EOG

Four electrodes including the ground electrode were placed on the forehead. Electrodes for the vertical channel were arranged vertically and electrodes for the horizontal channel were arranged horizontally to minimize interference between each channel. By sharing a positive electrode for the two channels, the number of electrodes was reduced. Although the amplitudes and signal qualities were lower than in conventional EOG, the EOG waveforms were well measured on the forehead. In the vertical channel, the amplitudes of the forehead EOG and conventional EOG were almost nine times different, and in the horizontal channel, they were almost three times different. For forehead EOG, the baseline drift was more severe than in conventional EOG. Baseline drift indicates the non-periodic DC-level fluctuation of the signal. Because the amplitude of the EOG signal in the time domain contains important information, fluctuations other than amplitude changes caused by eye movements have a negative effect on the EOG analysis. The excess baseline drift could be due to several factors, including lighting conditions, electrode contact, EMG artifacts, changes in skin potential, age, sex, or diurnal variations [19]. The waveforms similar to the conventional EOG were measured in the forehead, but the correlation coefficient between conventional EOG and forehead EOG was very low due to this baseline drift. The raw signals were differentiated to eliminate this baseline drift and then low-pass filtered to remove high-frequency noise. In this study, we have overcome this problem by differentiating the signal; however, in future research, it is necessary to study why the drift becomes worse in forehead EOG. After the signal processing, the correlation coefficients between conventional EOG and forehead EOG were 0.87 and 0.88 in vertical channel and horizontal channel, respectively. As shown in Figure 10, the “down” signal amplitude is smaller than that of the other movements. The reason for this is that the distance between the electrode and the source (dipole model described in introduction) is longer than the other movements. In the evaluation, no subject felt that it was difficult to control the downward movement because the threshold was adjusted to the amplitude of the “down”.

### 4.2. Comparison with Previous EOG Based HCI Studies

Conventional EOG-based HCI systems provide more directional movement detection than the system proposed in this study. In Wu’s study, they classified eight directional eye movements with 88.59% average accuracy (up, down, left, right, up-left, down-left, up-right, and down-right) [20]. In Phukpattaranont’s study, they proposed efficient features for classification of eye movements [21]. They classified not only the eight directional eye movements but also clockwise and counter clockwise eye movements.

Several studies measured EOG without using conventional electrode positions. Guo et al. developed a single-channel patchable EOG sensor which can be stuck on the forehead above the eyebrow [14]. They classified three types of eye movements (upward, downward, and blinking) with an 84% classification accuracy. In the study by Wu et al. they classified four classes of commands which were combined with three types of eye movements (double blink, single blink, and looking up) [15]. They measured single-channel EOG using a MindWave Mobile Headset (NeoroSky, San Jose, CA, USA), and the classification accuracy was 92.3%. An earphone-based eye gesture interface system was developed by Manabe et al. [18]. They classified three classes of eye gestures with three electrodes implanted in a pair of earphones. Belkacem et al. proposed an eye-movement-based communication system using two temporal EEG sensors with four electrodes [16]. They classified six classes of eye movements (left, right, up, down, blink, and center) with an 85.2% classification accuracy. In Zhang’s study, they proposed novel driving fatigue detection system using forehead EOG [17]. Although they did not present classification result of eye movement, their electrode position was similar to this paper. They placed four electrodes on the forehead, the reference electrode on the left mastoid, the ground electrode on the right mastoid. The advantage of our system over previous studies is that it detected six eye movements with a small number of electrodes located only on the forehead. In addition to the headband, electrodes placed on the forehead can be applied to many wearable devices such as cap and other head gears. And such wearable systems not only increase the usability of the user but also could reduce the resistance to the system. Table 2 summarizes the previous studies measuring EOG without using conventional electrode placement.

### 4.3. Classification Algorithm

We used the decision tree method to classify eye movements for a training-free algorithm. However because the amplitudes of the EOG signals were different for each user, a threshold setting for each user was required. In this study, to prevent unintended signaling due to gaze shifting when searching for the desired letter, the threshold was set to approximately 90% of the amplitude when the user moved their eyes intentionally. If the threshold is set too high, errors caused by normal gaze shifting can be reduced, but failures may occur due to decreasing amplitude caused by fatigue of the ocular muscle. If the threshold is set too low, failures can be reduced, but errors due to normal gaze shifting may increase. In the experiment using the modified Bremen speller, there was one error and five failures. The algorithm classified a “double blink” as “up (long blink)” twice because the interval threshold for classifying “blink” and “up (long blink)” was set too low. Thus, it is necessary to increase the interval threshold for users who cannot blink quickly. The five failures occurred because the threshold was set too high. There is a possibility that the failure rate may increase due to eye fatigue when used for experiments with a long duration. However, if the threshold is set too low, an undesired command can be signaled when the gaze shifts to find a letter. Therefore, it is important to set appropriate thresholds in this algorithm.

### 4.4. Applications

The Bremen BCI system, which was developed by Valbuena et al., is a virtual keyboard system using steady state visual evoked potential (SSVEP) [6]. The system has five flickering lights of different frequencies that induce SSVEP and encode four directional cursor movements and selection. Their typing speed was 2.56 LPM (re-computed in terms of their reported results). Although typing speeds were not as fast in this study, SSVEP-based BCI systems can increase speed by improving detection algorithms. Volosyak increased the typing speed of a Bremen BCI system to 8.28 LPM (re-computed in terms of their reported results) by applying a signal processing method [22]. SSVEP-based keyboards further increase the typing speed by increasing the number of stimuli. Hwang et al. developed a QWERTY style virtual keyboard using 30 LEDs flickering with different frequencies. Their average accuracy and typing speed were 87.58% and 9.39 LPM, respectively [23]. Furthermore, our previous study on hybrid SSVEP-P300 BCI achieved results of 93% accuracy and 7.15 LPM typing speed (re-computed in terms of reported results) [24].

In our system, the five flickering lights were removed and five commands were encoded by eye movements. In our experiments, a long blink was used to move the cursor upward instead of upward eye movement because the user found upward eye movement uncomfortable. Because of Bell’s phenomenon [25], the eyes moved upward when they were closed, so the same EOG waveforms were generated for both actions. As described earlier, the typing speed in the modified Bremen keyboard application was 10.81 LPM, which is higher than or equivalent to that of the recently proposed virtual keyboard mentioned above.

The modified Bremen speller cannot be used with a patient who cannot move their eyes in four directions. Thus, we tested our system with the virtual keyboard that uses automatic scanning, which required only one eye movement signal from patients who communicate using eye blinks. There are several types of scanning methods: element scanning, row-column scanning, and block scanning [26]. We chose row-column scanning method because we thought it was intuitive and suitable for a large number of targets as found on a keyboard. In this application, because a double blink was the encoded the switch that was used to select the row and column, only the vertical channel signal was used. As described in the results, the mean typing speed of row-column scanning was 7.75 LPM. The mean typing speed was lower than for the modified Bremen keyboard application, but the subjects reported that it was easier and more comfortable to use because it only used a double blink.

To prevent accidents caused by signaling errors, the power wheelchair driving was tested with the four classes of eye movements that the user felt most comfortable with. Although this test was conducted to evaluate the possibility of using forehead EOG for wheelchair driving and successful results were obtained, considering that the speed and the rotation angle of the wheelchair were fixed in the test environment for safety, more research is needed to make real world driving a possibility.

### 4.5. Limitations

There were several limitations in this study. First, the reaction times for left/right and up/down commands were different. Signals that were differentiated and low-pass filtered consisted of positive and negative peaks. For the ‘left’ and ‘up’ signals, the positive peak occurred before the negative peak. For the “right” and “down” signals, the negative peak occurred before the positive peak. Because the classification algorithm sampled the signal centered on the positive peak that exceed the threshold, a user could experience a delay when they performed “right” and “down” commands. In the virtual keyboard experiment, the subjects experienced some ambiguity in the training period but were able to quickly adapt; this did not affect the performance. In the wheelchair driving experiment, the subject had to move his eye beforehand, in order to consider the delay time. In future study, the algorithm must be modified to eliminate the delay as it confuses users and can be a big problem in wheelchair driving. It could be solved by simultaneously monitoring both positive and negative peaks. Second, the algorithm we proposed classified only 4 directional eye movements. As described in Section 4.2, several studies using conventional EOG provided more than eight directional movement classifications. However, unlike their studies, we used a forehead EOG, which has lower signal quality than conventional EOG. Thus, further work on signal processing and the classification algorithm are needed to detect more eye movements when using a forehead EOG. Third, we evaluated the proposed system with four subjects. It is necessary to evaluate with more subjects to confirm the generality. However, three subjects who were not familiar with the proposed system produced good results with little training, and this confirmed the possibility of a wearable forehead EOG system. It is important to note that the wearing configuration of the headband affected the performance of the system. Correct wearing of the headband is necessary because subjects may experience interference between both channels if the headband is worn incorrectly. A method of eliminating inter-channel interference through signal processing needs to be studied further.

## 5. Conclusions

We proposed novel electrode positions to detect eye movement on the forehead and developed a headband-type wearable forehead EOG measurement system. The system was evaluated and validated with two types of virtual keyboards and power wheelchair driving experiments operated by eye movements. This study could make a significant contribution to the literature because embedding EOG electrodes in a headband-type system could make it easier and more convenient for a disabled person to use assistive technology.

## Figures and Tables

**Table 1:** Results of virtual keyboard demonstrations.

	Modified Bremen Keyboard					Automatic RC Scanning Keyboard				
	S	E	F	Acc (%)	LPM	S	E	F	Acc (%)	LPM
S1	65	1	5	91.43	8.68	46	0	0	100	7.71
S2	70	7	1	89.74	9.20	46	0	1	97.87	7.71
S3	62	1	4	92.54	11.98	46	0	7	86.79	7.14
S4	63	2	4	91.30	13.37	46	0	2	95.83	8.42
Mean				91.25	10.81				95.12	7.75

S: Success, E: Error, F: Fail, Acc: Accuracy, LPM: Letters per minute.

**Table 2:** Comparison with previous studies that did not use conventional electrode placement.

Author	Electrodes	Channels	Classes	Accuracy	Electrode Positions	Correlation with Conventional EOG
Guo [14]	3	1	3	84.0%	Forehead	0.94 (Ch V)
Wu [15]	2	1	3	92.3%	Forehead, earlobe	-
Manabe [18]	3	1	3	-	In ears	-
Belkacem [16]	4	2	6	85.2%	Forehead, behind the ears, earlobe	-
Zhang [17]	6	2	-	-	Forehead, both mastoids	0.78 (Ch V), 0.85 (Ch H)
This paper	4	2	6	91.25%	Forehead	0.87 (Ch V), 0.88 (Ch H)

## Supplementary Materials

The following are available online at http://www.mdpi.com/1424-8220/17/7/1485/s1. Video S1: Forehead EOG demonstration #1—virtual keyboard, Video S2: Forehead EOG demonstration #2—virtual keyboard, Video S3: Forehead EOG demonstration #3—power wheelchair driving.
